# Supplementary material for: Polyketide synthase gene clusters in the ericoid mycorrhizal fungus Oidiodendron maius: genome-wide prediction, expression profiling, and functional characterization of knockout mutants
Source: Front Plant Sci. 2026 Sep 16;17:1924430. doi: 10.3389/fpls.2026.1924430 (PMC13623903; doi:10.3389/fpls.2026.1924430)
Supplement: Supplementary file 1 [file SupplementaryFile1.zip › Supplementary Materials/Supplementary Folder 1.zip!/Oidma1_AssemblyScaffolds_Repeatmasked/knownclusterblast/cluster51/jgi.p_Oidma1_35765_mibig_hits.html]

| MIBiG Protein | Description | MIBiG Cluster | MiBiG Product | % ID | % Coverage | BLAST Score | E-value |
| --- | --- | --- | --- | --- | --- | --- | --- |
| ADI24938.1 | VrtL | BGC0000168 | Viridicatumtoxin\_biosynthetic\_gene\_cluster | 59 | 98.0506822612 | 610 | 2.1e-174 |
| BAK26557.1 | putative\_MFS\_multidrug\_transporter | BGC0000977 | Cyclopiazonic\_acid\_biosynthetic\_gene\_cluster | 40 | 93.9571150097 | 374 | 1.9e-103 |
| ACD39772.1 | major\_facilitator\_superfamily\_transporter | BGC0000134 | Radicicol\_biosynthetic\_gene\_cluster | 40 | 98.8304093567 | 373 | 5.6e-103 |
| KFH48645.1 | putative\_transporter\_-like\_protein | BGC0000317 | Cephalosporin\_biosynthetic\_gene\_cluster | 37 | 93.1773879142 | 311 | 2.6e-84 |
| AGO65989.1 | putative\_MFS\_transporter | BGC0000992 | Fusaridione\_A\_biosynthetic\_gene\_cluster | 37 | 95.126705653 | 302 | 7.1e-82 |
| CAP95407.1 |  | BGC0001404 | Sorbicillin\_biosynthetic\_gene\_cluster | 33 | 92.9824561404 | 252 | 1.4e-66 |
| CCT67996.1 | bikaverin\_cluster-efflux\_pump | BGC0000030 | Bikaverin\_biosynthetic\_gene\_cluster | 31 | 91.6179337232 | 215 | 1.9e-55 |
| CCH26293.1 | putative\_MFS-type\_transporter | BGC0001158\_c4 | Bikaverin\_biosynthetic\_gene\_cluster | 30 | 94.5419103314 | 212 | 9.6e-55 |
| ABK64181.1 | MFS\_transporter | BGC0001247\_c4 | Cercosporin\_biosynthetic\_gene\_cluster | 31 | 92.2027290448 | 196 | 7.1e-50 |
| XP\_011392678.1 | hypothetical\_protein | BGC0001281 | Ustilagic\_acid\_biosynthetic\_gene\_cluster | 32 | 69.395711501 | 177 | 4.5e-44 |
| AMY15073.1 | MFS\_transporter | BGC0001339 | Squalestatin\_S1\_biosynthetic\_gene\_cluster | 29 | 41.910331384 | 84 | 3e-16 |
| AMY15055.1 | MFS\_transporter | BGC0001339 | Squalestatin\_S1\_biosynthetic\_gene\_cluster | 24 | 40.5458089669 | 74 | 4.1e-13 |
| AFU65888.1 | DacR2 | BGC0000216 | Dactylocycline\_biosynthetic\_gene\_cluster | 24 | 30.604288499 | 61 | 4.7e-09 |
| CAM56779.1 | Mem2 | BGC0000354 | Friulimicin\_biosynthetic\_gene\_cluster | 30 | 24.3664717349 | 59 | 1.8e-08 |
| WP\_016325506.1 | hypothetical\_protein | BGC0001283 | Arsenopolyketides\_biosynthetic\_gene\_cluster | 28 | 26.1208576998 | 55 | 2e-07 |
